# Competition of *Rhyzopertha dominica* and *Sitophilus oryzae* on six sorghum varieties

**DOI:** 10.1007/s11356-023-29807-9

**Published:** 2023-10-05

**Authors:** Paraskevi Agrafioti, Frank H. Arthur, Christos G. Athanassiou

**Affiliations:** 1 Laboratory of Entomology and Agricultural Zoology, Department of Agriculture Crop Production and Rural Environment, University of Thessaly, Phytokou Street, 38446 N. Ionia, Magnesia Greece; 2 U.S. Department of Agriculture, Agricultural Research Service, Center for Grain and Animal Health Research, 1515 College Avenue, Manhattan, KS 66502 USA

**Keywords:** Sorghum, Population growth, Grain infestation, Competition, Primary colonizers

## Abstract

We tested the effect of simultaneous infestation by adults of the lesser grain borer, *Rhyzopertha dominica* (F.) and the rice weevil, *Sitophilus oryzae* (L.) on six sorghum varieties. For this purpose, vials containing sorghum and either each species alone or both species were placed at 30 °C and 65% relative humidity. After ten days, all parental adults were removed and the vials were returned to the same conditions. Five weeks later the vials were emptied, to record adult emergence, the percentage of insect damaged kernels (IDK), and frass weight. Our results indicated that progeny production capacity for both species was not affected by the simultaneous presence of *R. dominica* and *S. oryzae*, and that adult emergence was more of a variety- mediated parameter. Both species had previously shown similar preferences towards specific sorghum varieties. IDK and frass were higher in vials containing *R. dominica* alone rather than *S. oryzae* alone, but these indicators were not always related to progeny production. Specifically, the most and the least frass production was noted on Sumac and PE sorghum varieties for both species, respectively. When the two species were placed together in the same vial, the most frass production was noted in the Non-Waxy Burgundy and Sumac varieties. Our results suggest that varietal resistance in sorghum could be utilized to help reduce post-harvest infestations by *S. oryzae* and *R. dominica*.

## Introduction

Sorghum is one of the most important grains globally, and is considered as one of the promising alternative cereals to wheat, rice and maize (Cardoso et al. 2017). Although there are different species of sorghum, the vast majority of the published reports are for *Sorghum bicolor* L., known also as milo, which is cultivated for both food and feed, but also for silage, in combination with other crops (Kangama and Rumei 2005). Sorghum is considered as a highly nutritious grain, rich in polyphenols and flavonoids, while its production has gradually increased during the last decade (Cardoso et al. 2017). Several studies documented that sorghum can be infested by different insect pests during storage that can cause serious damage and qualitative degradations (Arthur et al. 2020; Gourgouta et al. 2021; Lampiri et al. 2020, 2023). For instance, Lampiri et al. (2020) reported that larvae of the khapra beetle, *Trogoderma granarium* Everts (Coleoptera: Dermestidae) could develop on different sorghum milling fractions, with sorghum bran being most suitable for development.

Varietal resistance could be a key factor in stored product protection, and should be taken into account in post-harvest integrated pest management (IPM) protocols (Throne et al. 2000). In this context, selecting the right variety/hybrid may result in less infestation by stored product insects, which could reduce input from insecticides. There are several published studies on varietal resistance for other grains, particularly wheat, rice and maize. For instance, Fang et al. (2002) found that reproduction of the lesser grain borer, *Rhyzopertha dominica* (F.) (Coleoptera: Bostrychidae) and the rice weevil, *Sitophilus oryzae* (L.) (Coleoptera: Curculionidae) was higher on certain classes of wheat, suggesting that some physicochemical characteristics, such as kernel hardness and size, play a key role in the progeny production capacity of stored product insect species. Moreover, Kavallieratos et al. (2010) compared the development of *R. dominica, S. oryzae* and the confused flour beetle, *Tribolium confusum* Jacquelin du Val (Coleoptera: Tenebrionidae) on three wheat varieties, Athos, Sifnos and Pontos, and found that progeny production of these species was always higher in Pontos, as compared with the other two varieties. Similarly, in rice, Doherty et al. (2023), did an extensive screening of varietal resistance on different rice varieties for *S. oryzae*, and found that resistance of adults followed different patterns in comparison to larvae. Due to the increased importance of sorghum, varietal resistance in sorghum in its post-harvest stages has been also examined during the last years, but there are still disproportionally few data as compared to other major grains. Recently, Gourgouta et al. (2021) tested four sorghum varieties for their susceptibility from *T. granarium* infestation, and found that the progeny production capacity of this species was similar in all four varieties tested, but the speed of development varied with variety.

Coexistence of stored product insect species in the same commodity has been long regarded as a model to evaluate the fundamental principles of competition among organisms that share the same environment (Crombie 1945; Birch 1945a,b; Giga and Canhao 1993; Nansen et al. 2009; Athanassiou et al. 2014, 2017). Almost eighty years ago, Birch (1945a,b) studied the development of two of the most important primary colonizers of grain *R. doninica* and *S. oryzae* in relation with temperature, and indicated that this coexistence may continue for longer periods, despite the fact that both species utilize the same part of the kernel. However, more recent studies have shown that colonization of a certain stored product insect species may result in the rapid extinction of the other species on grain, especially in the case of primary colonizers (Giga and Canhao 1993; Athanassiou et al. 2017; Kavallieratos et al. 2017). For instance, Kavallieratos et al. (2017) found that at elevated temperatures, *T. granarium* could rapidly outcompete both *S. oryzae* and *R. dominica* on wheat, and, to a lesser extent, on rice. Similar results have been also reported in the case of stored product psocids, where *Liposcelis bostrychophila* Badonnel (Psocoptera: Liposcelididae) could outcompete conspecifics in stored grains (Athanassiou et al. 2014). Moreover, Sakka and Athanassiou (2018) found that on maize, the larger grain borer, *Prostephanus truncatus* (Horn) (Coleoptera: Bostrychidae) had a higher population growth than the yam beetle, *Dinoderus porceilus* (Lesne) (Coleoptera: Bostrychidae) or *R. dominica*. Conversely, in the same study, it was found that on wheat, only the two latter species were able to produce offspring, while *P. truncatus* was unable to reproduce (Sakka and Athanassiou 2018). Moreover, in a study where the competition of *S. oryzae* with the maize weevil, *Sitophilus zeamais* Motschulsky and the granary weevil, *Sitophilus granarius* (L.), Athanassiou et al. (2017) found that population growth was different on maize as compared with rice. All the above suggest that the outcome of the competition of stored product beetles is commodity-mediated, and that the domination of one species to one single commodity may not reassure that the same species can be also dominant in a different host. Nevertheless, to our knowledge, there are no data available for the competition of primary colonizers on sorghum. Hence, in our study, we tested competition of *S. oryzae* and *R. dominica* on different sorghum varieties, in an attempt to combine both aspects: competition of two primary colonizers and varietal resistance.

## Materials and methods

### Tested insects

The insects that were tested in our experiments were *R. dominica* and *S. oryzae*. These colonies have been maintained for more than 30 years in the Laboratory of Stored Product Insect and Engineering Research (SPIERU), in Manhattan Kansas State. The above colonies have not been exposed to pesticides, so they are considered susceptible colonies. The rearing media for each species were 90% whole wheat and 10% cracked wheat for *R. dominica* and hard wheat equilibrated to 13.5% moisture content for *S. oryzae*. The colonies are kept in environmental growth chamber in the laboratory at 28.0 ± 0.5°°C and 60 ± 5% moisture content with a photoperiod of 12:12 h (L:D).

### Sorghum varieties

Six sorghum varieties were examined in this study: Waxy Burgundy, Non-Waxy Burgundy, White, Sumac, PE, SC and were provided from the USDA-Agricultural Research Service- Center for Grain and Animal Health Research (CGAHR), in Manhattan, Kansas, USA. All varieties were free of pesticides and uninfested with 12.3% moisture. These sorghum varieties were referred in Arthur et al. (2020) as red waxy, red non-waxy, white and red non-tannin, respectively.

### Experimental procedure

Plastic cylindrical vials 3 cm in diameter and 8 cm high (177 ml volume, Thornton Plastics, Salt Lake City, UT, USA) were used in this study. The vials were filled with 30 g of each sorghum variety in separate vials. Ten adults of either *R. dominica*, *S. oryzae* or both (i.e., 10 adults of *R. dominica* and 10 adults of *S. oryzae*, which means 20 adults) were placed into each individual vial containing one of the six sorghum varieties. All vials were placed in incubator (Percival Scientific, Perry, IA, USA) set at 30 °C and 65% relative humidity (r.h.), in continuous darkness, with separate vials for each species and for each sorghum variety. There was a series of vials for each sorghum variety. For each combination, there was a series of three replicates, and the entire procedure was repeated two times (3 replicates with two sub-replicates = 6 vials were used for each combination).

After 10 days, the contents of each vial were emptied separately into white enamel laboratory pan and the parental adults were removed using forceps, then discarded. The contents of each vial (sorghum kernels and any frass produced by feeding of the parental adults) were then returned to the vial, and the vials in turn returned to the incubator. After 5 weeks, the vials were removed from the incubator. The contents of each vial containing either were emptied into a series of two 20.3 cm brass circular diameter sieves (Dual Manufacturing Company, Franklin Park, IL, USA). The top sieve was either a #10 sieve (2 mm mesh openings) or a #12 sieve (1.77 mm mesh openings). The #10 sieve was needed to retain the sorghum kernels while allowing the *S. oryzae* to fall through the mesh openings, while the #12 sieve was used to retain kernels and allow the *R. dominica* to fall through the mesh openings The next sieve was a #30 sieve with 0.60 mm openings to retain both species, and allow the frass to fall through the mesh openings to the pan, which had a solid bottom to catch the frass. The progeny adults from each vial were then emptied from the sieve, then counted and recorded, and afterwards discarded. The frass in each vial was then weighed by emptying the contents of the sieve onto a piece of weighing paper, which was then placed in a Mettler 4000 PC Balance (Mettle-Toledo, Toledo, OH, USA) to obtain the weight of the frass material. To obtain a value for the percentage of insect damaged kernels (IDK), 100 kernels from each vial were selected from the top sieve and placed in a plastic Petri dish (bottom area of 62 cm^2^) and examined individually under a stereo microscope to look for the emergence hole made by the progeny adult when it exited the kernel. When an emergence hole was noted, the kernel was classified as an IDK, and data were reported as a percentage. After the IDK counts were made sorghum kernels from the vial were discarded.

### Statistical analysis

The statistical analyses were conducted using JMP 8 software (SAS Institute Inc., Cary, NC). Data were initially submitted to homoscedasticity and were assessed for assumption of normality by using Levene’s test. Where the variances were not equal, the data were transformed to Log + 1. Then, the data of the number of individuals (population growth) and grain parameters (insect damaged kernel and frass) were analyzed by using ANOVA with insect species and sorghum varieties, as the main effects. Subsequently, for the number of individuals (population growth), insect damaged kernels and frass one-way ANOVAs were performed, for each insect species and for both species, in order to highlight the differences among the tested sorghum varieties. The means were separated using the Tukey–Kramer (HSD) test at *P* < 0.05. For vials in which the species were together, the data for number of individuals Student’s *t*-test were performed, in order to indicate the differences between the two species for each sorghum variety.

## Results

Population growth (number of individuals), for both species (*R. dominica* and *S. oryzae*) was significant with respect to variety (*P* < 0.001) (Table 1). When each species alone or with the other species on the different sorghum varieties, the effect (alone/both) and the interaction (varieties * alone/both) were not significant (Table 1). With insect damaged kernels (IDK), for both species all main effects and the interaction were significant (*P* < 0.001) (Table 2). For frass production for both species, all main effects and the interaction were significant, with only one exception (varieties by alone/both) of *R. dominica,* since *P* = 0.215 (Table 3).

**Table 1:** ANOVA parameters for the individuals for main effects and associated interactions of *Rhyzopertha dominica* and *Sitophilus oryzae*, at different sorghum varieties

		*Rhyzopertha dominica*		*Sitophilus oryzae*	
	*df*	F	*P*	F	*P*
Whole model	11	14.51	< 0.001	15.27	< 0.001
Intercept	1	331.30	< 0.001	384.61	< 0.001
Varieties	5	31.51	< 0.001	30.35	< 0.001
Alone/Both	1	1.01	0.317	3.46	0.067
Varieties * Alone/Both	5	0.21	0.956	2.55	0.036

For *R. dominica*, O’Brien test was used: F = 1.581, *P* = 0.195 and for *S. oryzae* O’Brien was used: F = 1.899,* P* = 0.124

**Table 2:** ANOVA parameters for Insect Damaged Kernels (IDK), for all main effects and associated interactions for *Rhyzopertha dominica* and *Sitophilus oryzae,* at different sorghum varieties

		*Rhyzopertha dominica*		*Sitophilus oryzae*	
	*df*	F	*P*	F	*P*
Whole model	11	19.97	< 0.001	18.94	< 0.001
Intercept	1	476.73	< 0.001	543.21	< 0.001
Varieties	5	29.47	< 0.001	30.17	< 0.001
Alone/Both	1	47.21	< 0.001	31.63	< 0.001
Varieties * Alone/Both	5	5.03	< 0.001	5.18	< 0.001

For *R. dominica*, Levene’s test was used: F = 2.238, *P* = 0.076 and for *S. oryzae* after the transformation data (Log + 1) O’Brien test was used: F = 2.070, *P* = 0.095

**Table 3:** ANOVA parameters for Frass production, for all main effects and associated interactions for *Rhyzopertha dominica* and *Sitophilus oryzae*, at different sorghum varieties

		*Rhyzopertha dominica*		*Sitophilus oryzae*	
	*df*	F	*P*	F	*P*
Whole model	11	20.90	< 0.001	27.06	< 0.001
Intercept	1	442.87	< 0.001	333.97	< 0.001
Varieties	5	41.87	< 0.001	29.42	< 0.001
Alone/Both	1	13.29	< 0.001	100.89	< 0.001
Varieties * Alone/Both	5	1.46	0.215	9.94	< 0.001

For *R. dominica,* O’Brien test was used: F = 1.019, *P* = 0.424 and for *S. oryzae* O’Brien test was used: F = 1.303, *P* = 0.289

### Population growth

**Fig. 1:**
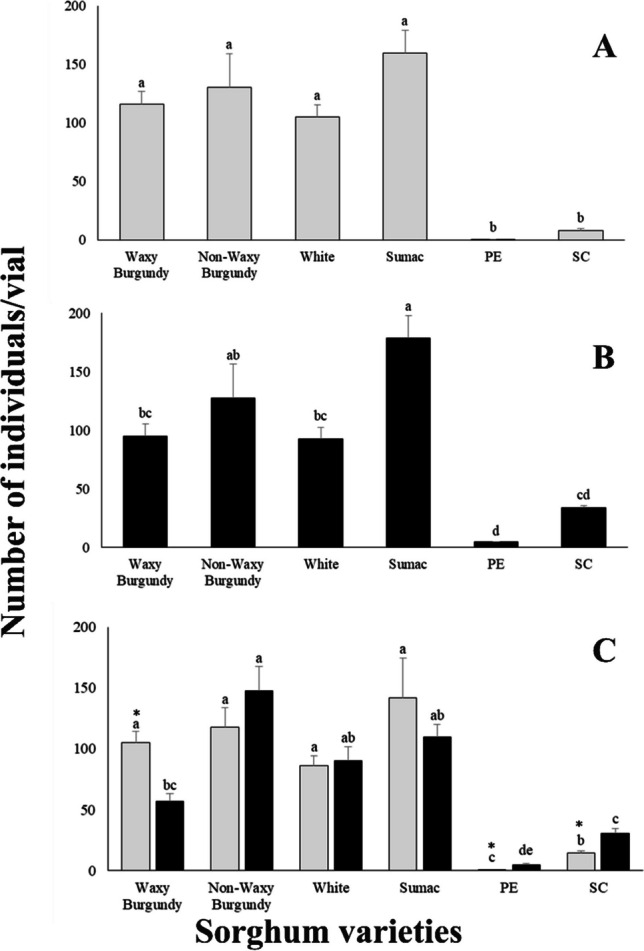
Mean ± SE of population growth for *Rhyzopertha dominica* (**A**), *Sitophilus oryzae* (**B**) and both (*Rhyzopertha dominica* and *Sitophilus oryzae*) (**C**) on six sorghum varieties. For each species, means followed by the same lowercase letter are not significantly different (Tukey–Kramer HSD test at 0.05). Where no letter exist, no significant differences were noted. ANOVA parameters for *R. dominica* (**A**) were: F = 18.485,* P* < 0.001 and for *S. oryzae* (**B**) were: F = 13.527, *P* < 0.001. Within each sorghum variety (**C**), after transformation data Brown-Forsythe test was used for both species, for *R. dominica*: F = 1.394, *P* = 0.254, and for *S. oryzae*: F = 2.379, *P* = 0.062. ANOVA parameters for (C) *R. dominica*: F = 29.716, *P* < 0.001 and for *S. oryzae*: F = 55.215, *P* < 0.001. Means with asterisks (*) indicate significant differences between the two species (*R. dominica* and *S. oryzae*) tested, according to Student’s test at 0.05. *T*-test parameters were for Waxy-Burgundy: t = 4.291, *P* < 0.001, for PE: t = 3.210, *P* < 0.001 and SC: t = 3.65, *P* < 0.001

In all cases, the lowest number of individuals was recorded on the PE sorghum variety and the highest number of individuals was recorded on Sumac variety (Fig. 1). For *R. dominica* and *S. oryzae*, significant differences were noted among all sorghum varieties (Fig. 1A, B). Moreover, when the two species were placed together in the same vial, significant differences were observed among all varieties (Fig. 1C). Additionally, for *S. oryzae* population growth was slightly reduced in Waxy Burgundy when the two species were placed in the same vial (Fig. 1B, C). In case of *R. dominica*, a similar number of progeny was produced on Waxy Burgundy variety placed alone or together in the vials (Fig. 1A, C). Significant differences were recorded between the two species tested on three sorghum varieties (Waxy Burgundy, PE, SC) (Fig. 1C).

### Insect Damaged Kernels – IDK

**Fig. 2:**
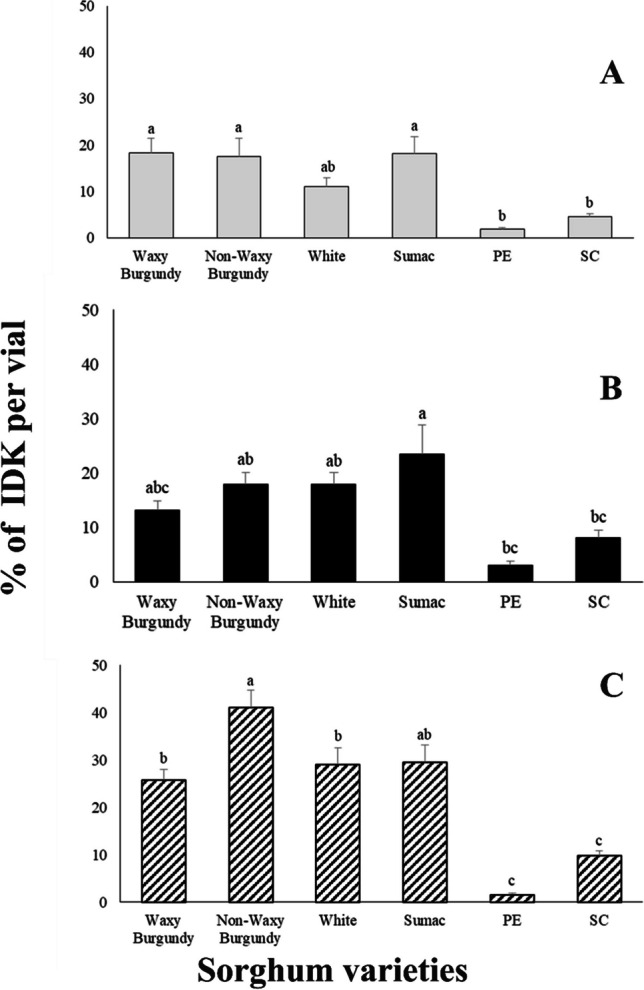
Mean (percentage ± SE) of Insect Damaged Kernels (IDK) for *Rhyzopertha dominica* (**A**), *Sitophilus oryzae* (**B**) and both (*Rhyzopertha dominica* and *Sitophilus oryzae*) (**C**) on six sorghum varieties. For each species, means followed by the same lowercase letter are not significantly different (Tukey–Kramer HSD test at 0.05). Where no letter exist, no significant differences were found. ANOVA parameters for *R. dominica* (**A**) were: F = 7.160, *P* < 0.001, for *S. oryzae* (**B**) were: F = 14.388, *P* < 0.001 and for (**C**) were: F = 26.911, *P* < 0.001

In general, there were significant differences among the different sorghum varieties regarding the percentage of IDK (Fig. 2). The lowest percentage of IDK were found on the PE (1.6 to 35%) and SC varieties (4.6 to 9.8%) varieties (Fig. 2). The percentages of IDK for *R. dominica* and *S. oryzae,* followed the same pattern in vials where the species were placed alone (Fig. 2A, B). The percentages of IDK was much higher in most of the sorghum varieties tested when both species were present (Fig. 2C). Additionally, the highest percentages of IDK were in Non-Waxy Burgundy (41.1 ± 3.6%) (Fig. 2C).

### Frass production

**Fig. 3:**
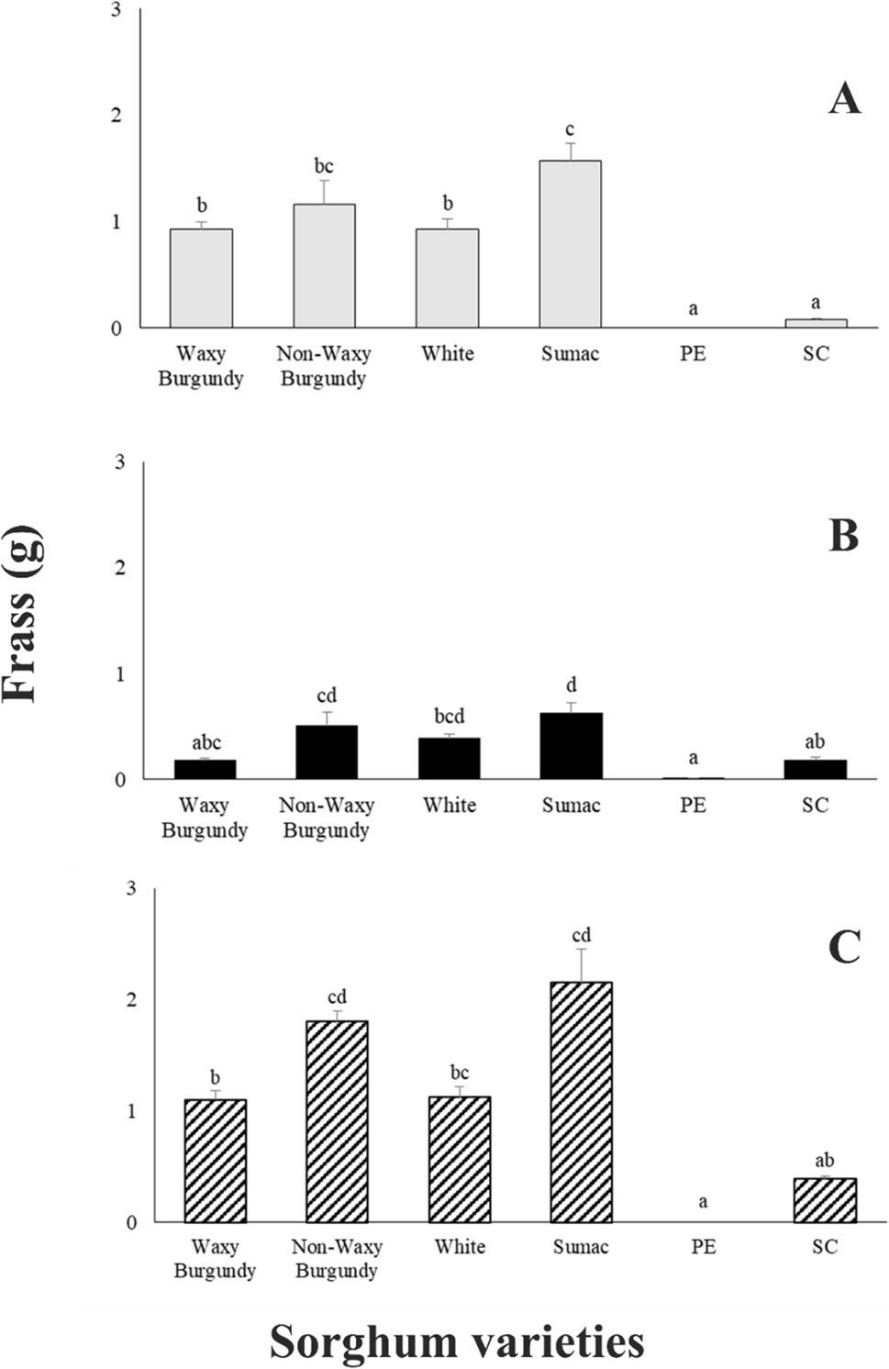
Mean ± SE of Frass for *Rhyzopertha dominica* (**A**), *Sitophilus oryzae* (**B**) and both (*Rhyzopertha dominica* and *Sitophilus oryzae*) (**C**) on six sorghum varieties. For each species, means followed by the same lowercase letter are not significantly different (Tukey–Kramer HSD test at 0.05). Where no letter exist, no significant differences were found. ANOVA parameters for *R. dominica* (**A**) were: F = 21.817, *P* < 0.001, for *S. oryzae* (**B**) were: F = 9.319, *P* < 0.001 and for (**C**) were: F = 21.586, *P* < 0.001

In general, the levels of frass production were lowest in PE and SC sorghum varieties (Fig. 3). Nevertheless, in most of the cases, where species were present alone or together in the vials, there were significant differences in frass production among the sorghum varieties (Fig. 3). Moreover, more frass was recorded in vials that contained *R. dominica*, either alone or with *S. oryzae*, as compared to the vials that contained *S. oryzae* alone (Fig. 3). Additionally, the most frass was observed in the Non-Waxy Burgundy and Sumac varieties, especially when the two species were placed together in the same vial (Fig. 3C).

## Discussion

Our findings clearly demonstrate that there are noticeable differences in the susceptibility level of the six sorghum varieties tested regarding potential for population growth and infestation patterns of *S. oryzae* and *R. dominica.* Moreover, we found that population growth was higher on Sumac than in the other sorghum varieties, despite the fact that significant differences were not always recorded. However, soft wheat could be considered as a more preferred commodity than the tested sorghum varieties. Gourgouta et al. (2021) have shown that soft wheat was a very good food source for the population growth of *T. granarium*, as compared to different sorghum varieties. Nevertheless, in that study, the authors found that the highest number of individuals of *T. granarium* was found on White sorghum and the lowest on Sumac, which was different than the susceptibility patterns found here for *R. dominica* and *S. oryzae*.

In a recent study, Arthur et al. (2020) compared the susceptibility of Sumac, White, Non-waxy Burgundy and Waxy-Burgundy to infestation by *R. dominica* and reported that there were some variety characteristics that could play a significant role in progeny production capacity, while offspring of *R. dominica* could have been linked with physical characteristics of the grain kernels, such as kernel hardness and kernel weight. In our study, population growth of *R. dominica* was generally higher on Sumac and Non-waxy Burgundy, which could be attributed to the fact that these two varieties have low starch and high protein content, which seems to be a preferable characteristic for the development of *R. dominica* (Arthur et al. 2020).

Interestingly, the coexistence of the species tested in the same vial had little effect on their population growth. In general, the rank of the different varieties in terms of adult emergence did not change much even were both species were present in the same vial, which is particularly important, as larvae of both species utilize the internal part of the sorghum kernel. Our results show that the overall progeny production was slightly reduced in the case where both species were present, but to a proportional degree for both species. For instance, more than 160 *R. dominica* adults/vial were detected in vials containing Sumac when the species was alone, while when this species was in the same vial with *S. oryzae*, this figure was close to 140 *R. dominica* adults/vial. For the same variety, the presence of *R. dominica* in the vials that contained *S. oryzae* adults affected adult emergence of the latter species, as compared with the vials containing *S. oryzae* alone. This suggests that *S. oryzae* was affected more by the simultaneous presence of *R. dominica* than vice versa. Conversely, in the case of Non-Waxy Burgundy, the presence of *R. dominica* increased *S. oryzae* progeny production, as compared with the presence of *S. oryzae* alone. All the above support our observation that the outcome of this competition is more variety-mediated, rather than species- mediated. A previous work by Athanassiou et al. (2017) showed that progeny production of both *S. oryzae* and *S. zeamais* was affected by the type of the commodity, but the outcome of the competition was literally the same, with *S. oryzae* having a slight supremacy over *S. zeamais* in both commodities. However, our experimental scenario was largely based on the theory of unlimited provision of food sources, which allowed both species to develop high population densities, which were comparable to each other. The continuance of this experiment in the same initial sorghum quantities, e.g. for one more generation, is likely to reveal the superior and the inferior competitor, due to stress in the scarcity of food. Similar results have been reported in a previous work where under conditions of limited food availability *L. bostrychophila* dominated over other stored product psocid species (Nansen et al. 2009; Athanassiou et al. 2014). Moreover, in our scenario both species “arrived” on the grain the same time in similar parental adult densities, which may not be realistic in “real world” conditions. In a recent study, Baliota et al. (2021) found that one of the fundamental factors in the outcome of the competition between *P. truncatus* and *S. oryzae* was the time that each species starts infesting the grain, indicating that the first colonizer usually has an advantage.

Apart from the population growth, we have recorded that the simultaneous presence of both species resulted in an increase in grain damage. Although we are unaware of the species that infested most of the kernels, patterns presented here show that the number of IDK when both species were present, was close to the sum of the IDK numbers that has been produced by each species alone, regardless of the variety. This is particularly evident in the case of frass, where, considering the overall data, we recorded that frass production caused by *R. dominica* was higher than that of *S. oryzae*. In general, the presence of frass is considered as beneficial for the development of stored product Bostrychidae, such as *R. dominica* or *P. truncatus* (Edde 2012; Quellhorst et al. 2021). However, the infestation patterns that were noted here were not proportional of the progeny production capacity of the two species, as although *R. dominica* caused more grain damage, adult emergence was comparable with that of *S. oryzae*. This characteristic has been also reported in previous studies studying competition of primary colonizers in stored grains (Baliota et al. 2021; Quellhorst et al. 2020, 2021).

The results of the present study show that certain sorghum varieties are resistant to infestation by both species tested, and should be considered further for this purpose. Our results support the data that are presented by Arthur et al. (2020) for the specific physicochemical characteristics of sorghum varieties in the infestation by *R. dominica*, and indicate that these characteristics may have a similar effect in the case of *S. oryzae* as well. Furthermore, we found that the simultaneous presence of both species in sorghum does not alter their progeny production capacity, at least for the incubation interval tested here.

## Data Availability

The authors confirm that the data supporting the findings of this study are available within the article.
